# A chromosome scale assembly of the tarnished plant bug, *Lygus lineolaris* (Palisot de Beauvois), genome

**DOI:** 10.1186/s13104-023-06408-w

**Published:** 2023-06-27

**Authors:** O. P. Perera, Surya Saha, James Glover, Katherine A. Parys, K. Clint Allen, Snejana Grozeva, Ryan Kurtz, Gadi V. P. Reddy, J. Spencer Johnston, Mark Daly, Thomas Swale

**Affiliations:** 1 Southern Insect Management Research Unit, USDA ARS, 141 Experiment Station Road, Stoneville, MS 38776 USA; 2 Boyce Thompson Institute, 533 Tower Rd, Ithaca, NY 14853 USA; 3 Pollinator Health in Southern Crop Ecosystems Research Unit, USDA ARS, 141 Experiment Station Road, Stoneville, MS 38776 USA; 4 Institute of Zoology, Bulgarian Academy of Sciences, 1 Tsar Osvoboditel, Sofia, 1000 Bulgaria; 5 Cotton, Incorporated, Cary, NC 27513 USA; 6 Department of Entomology, Texas A&M University, College Station, TX 77843 USA; 7 Dovetail Genomics, LLC, 100 Enterprise Way, Suite A101, Scotts Valley, CA 95066 USA

**Keywords:** *Lygus lineolaris*, Tarnished plant bug, Miridae, Genome assembly, Cotton Pest

## Abstract

**Objective:**

The tarnished plant bug (TPB), *Lygus lineolaris* (Palisot de Beauvois) (Hemiptera: Miridae), is a pest damaging many cultivated crops in North America. Although partial transcriptome data are available for this pest, a genome assembly was not available for this species. This assembly of a high-quality chromosome-length genome of TPB is aimed to develop the genetic resources that can provide the foundation required for advancing research on this species.

**Results:**

The initial genome of TPB assembled with paired-end nucleotide sequences generated with Illumina technology was scaffolded with Illumina HiseqX reads generated from a proximity ligated (HiC) library to obtain a high-quality genome assembly. The final assembly contained 3963 scaffolds longer than 1 kbp to yield a genome of 599.96 Mbp. The N50 of the TPB genome assembly was 35.64 Mbp and 98.68% of the genome was assembled into 17 scaffolds larger than 1 Mbp. This megabase scaffold number is the same as the number of chromosomes observed in karyotyping of this insect. The TPB genome is known to have high repetitive DNA content, and the reduced assembled genome size compared to flowcytometric estimates of approximately 860 Mbp may be due to the collapsed assembly of highly similar regions.

**Supplementary Information:**

The online version contains supplementary material available at 10.1186/s13104-023-06408-w.

## Introduction

The tarnished plant bug (TPB), *Lygus lineolaris* (Palisot de Beauvois) (Hemiptera: Miridae), has a broad host range exceeding 300 plant species including a large number of cultivated crops in the United States [1, 2]. TPB has five nymphal stages and the ovipositor in the center of abdominal sternites in adult females can distinguish females from males (Supplementary Fig. S1). TPB is present in the continental United States, Canada, and Mexico. This pest causes significant economic damage to a diversity of vegetable crops, fruits, and nursery stock including strawberries, cotton, and seedlings of conifers [3–7]. In 2020, TPB infested more than 4.8 million acres of cotton resulting in an estimated $157 million in control costs and yield losses [8]. Current control of TPB in cotton is carried out almost exclusively using synthetic insecticide sprays. Formulations and mixtures of insecticides including carbamates, organophosphates, nicotinamides, neonicotinoids, and pyrethroids are routinely used along with an insect growth regulator (novaluron) to manage TPB in commercial agriculture. A systemic insecticide (sulfoxaflor) is permitted under special conditions to manage TPB in cotton. Insecticide resistance in the TPB has been reported in the Mississippi Delta since 1995 [9–11]. As in the case of many insects, the susceptibility of TPB to different chemicals within three major classes of insecticides (carbamate, organophosphate, and pyrethroid) commonly used for pest control in cotton has varied over the past forty years [12]. Most insecticides that previously provided good control of TPB currently exhibit diminishing effectiveness [13, 14].

Resistance to insecticides may develop by breaking down of the insecticides through a range of mechanisms: by increased levels or enhanced activity of detoxifying enzymes (metabolic resistance), by resisting the binding of the chemical through genetically modified target sites (target-site resistance), by changing the properties of the exoskeleton to reduce the rate of penetration of contact insecticides (penetration resistance), or through behavioral resistance by developing the ability to detect insecticides and avoid exposure [15–18]. These adaptations reflect shifts in frequencies of alleles responding to changing environmental conditions by substituting genes in populations over time [19, 20]. These shifts in allele frequencies of genes responding to environmental factors can be identified by monitoring the populations using genetic markers. The number of insect population genomic studies has rapidly increased recently due to the availability of genomic data and cost-effective, high throughput sequencing methods used to generate data [reviewed in: 21, 22]. Navel orangeworm, *Amyelois transitella* [23], brown planthopper, *Nilaparvata lugans* [24], and Asian tiger mosquito, *Aedes albopictus* [25], for example, are among the subjects of a growing number of recent population genomic studies. However, lack of genome sequence data has precluded population genomic studies of *Lygus* species.

Despite TPB being a pest of several economically important crops grown in North America, apart from a few population genetic and transcriptome and gene expression studies, there is a general paucity of research on the genetics of TPB [26–31]. Therefore, the development of a comprehensive set of genetic resources including a high-quality genome, full transcriptome with an official gene set that identifies all isoforms, and genetic markers suitable for population genomic and quantitative genetic studies is needed for this species. Community insect genomics initiatives like the i5k consortium [32] and more recently Ag100Pest [33] and AgriVectors [34] have also highlighted the far-reaching consequences and benefits of creating reference-grade genomics resources and building open access tools to make them available [34, 35]. Our goal for sequencing the genome of TPB was to develop these genetic resources that will significantly advance genetics research on TPB. This will allow us to identify the candidate detoxification gene repertoire and genetic polymorphisms required for genetic mapping and ecological genetic studies in the TPB.

## Results and discussion

**Fig. 1:**
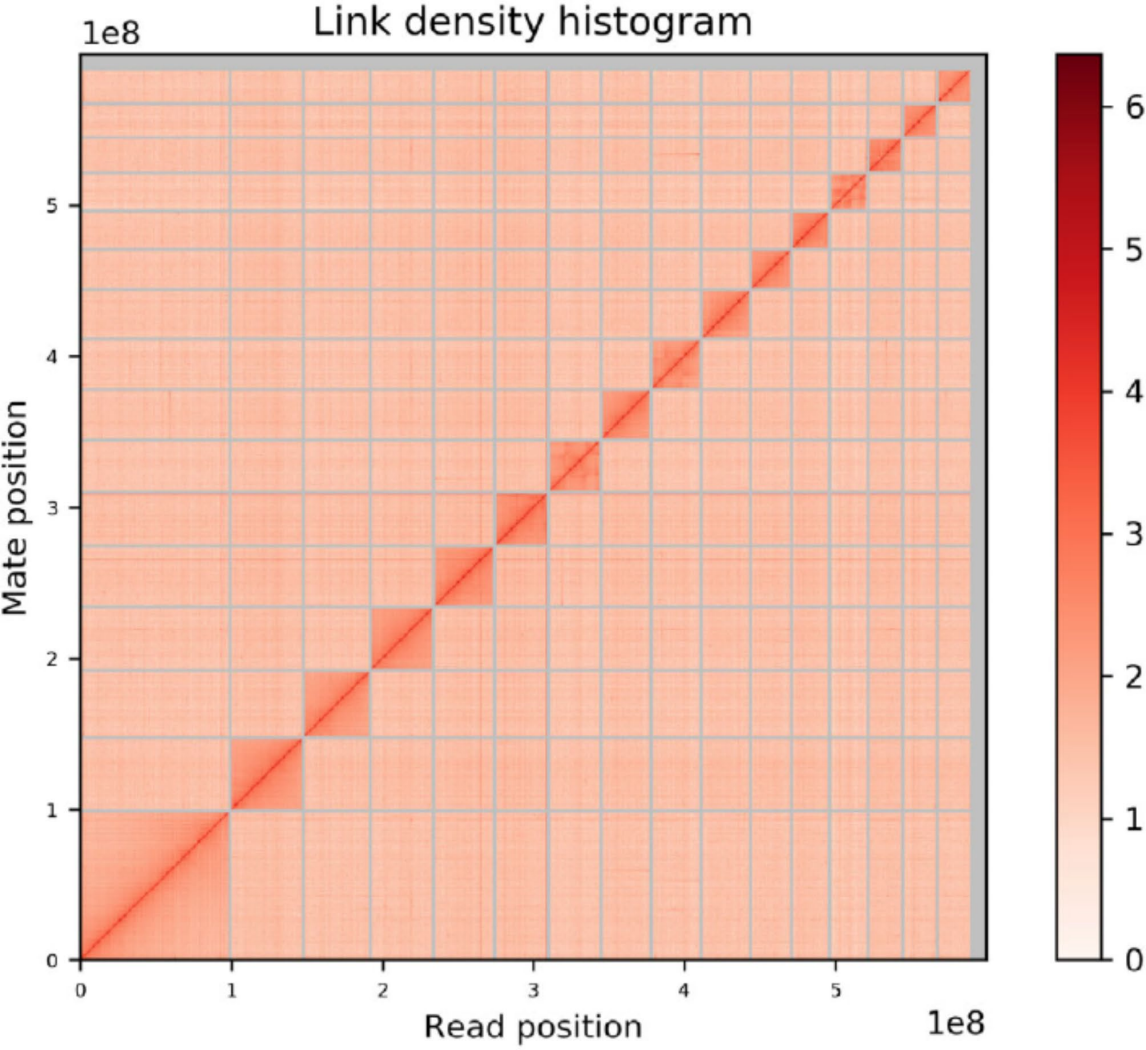
Linkage density histogram of *Lygus lineolaris* genome assembly generated from HiC read pairs. The first and second read in a read pair grouped into bins is plotted in the x and y axes, respectively. The intensity of color of each square represents the number of read pairs in each bin. Scaffolds smaller than 1 Mb were not used in this histogram

The Meraculous Assembler [36] estimated the genome to be approximately 800 Mb. The scaffolded Illumina-only assembly with two Illumina libraries followed by a round of scaffolding with a third Illumina library was 599.96 Mb with a N50 of 19.8Kb and L50 of 7.1Kb. The total coverage of the *L. lineolaris* genome by the three Illumina paired-end libraries was 240X. Long range Hi-C scaffolding connected the scaffolds from the Meraculous assembly to create the final assembly with 3963 scaffolds with an N50 of 35.64 Mbp and a total length of 600 Mb (Fig. 1). This assembly contains 80 Mb of Ns with 13.4Kb of Ns per 100Kb of genomic sequence. Accession numbers for genome sequence data are given in the Table 1. This whole genome sequencing project has been deposited at DDBJ/ENA/GenBank under the accession JAEMON000000000. The version described in this paper is version JAEMON010000000.

**Table 1:** Database accession numbers for nucleotide sequence reads and the genome assembly of *Lygus lineolaris* and the supplementary data files deposited in the Figshare database (www.figshare.com)

Sample Name	Description	File Type	Accession
DTG_HiC_1196	*L. lineolaris* Hi-C scaffolding (Omni-C library)	Single Illumina miSeq reads	SRR13721411
DTG_HiC_1179	*L. lineolaris* Hi-C scaffolding (Omni-C library)	Paired-end Illumina miSeq reads	SRR13721412
DTG-OmniC-56	* L. lineolaris* Hi-C scaffolding (Omni-C library)	Paired-end Illumina reads	SRR13721413
DTG-OmniC-55	* L. lineolaris* Hi-C scaffolding (Omni-C library)	Paired-end Illumina HiSeq reads	SRR13721414
Index_12.CP-3809	* L. lineolaris* WGS (Chicago Library)	Paired-end Illumina HiSeq reads	SRR13721415
Index_6.CP-3770	* L. lineolaris* WGS (Chicago Library)	Paired-end Illumina HiSeq reads	SRR13721416
CP-2092_S2	*L. lineolaris* WGS (Shotgun Library)	Paired-end Illumina HiSeq short reads	SRR13721417
Genome Assembly	*L. lineolaris* draft genome assembly version 1.0	Genome assembly (Fasta)	PRJNA589321; SAMN13280589 PRJNA685878; SAMN17087946
Processed Genome Assembly	*L. lineolaris* draft genome assembly version 1.0	NCBI WGS object	JAEMON010000000
Supplementary Data Tables	Table S1; Table S2	MS Excel	DOI:10.6084/m9.figshare.23302250
Supplementary Figures	Figure S1, Figure S2	Adobe PDF	DOI:10.6084/m9.figshare.23313344

BUSCO [37] evaluation of the completeness of the Illumina and Hi-C assemblies based on the Hemipteran (2510) and Arthropoda (1013) marker sets indicated that the Hi-C scaffolded assembly improved over the short-read Illumina assembly with an 85.1% of the complete assembly. Only 5.6% of the 1013 Arthropoda BUSCO markers missing (Table 2).

**Table 2:** BUSCO completeness statistics for the Lygus lineolaris genome assembly with Arthropoda and Hemiptera marker set. BUSCO version 5.2.2 was used to generate these statistics

BUSCO Database	Complete BUSCOs	Complete and single-copy BUSCOs	Complete and duplicated BUSCOs	Fragmented BUSCOs	Missing BUSCOs	Total BUSCO groups searched
Illumina Hemiptera	52.4	52.0	0.4	0.3	47.3	2510
Hi-C Hemiptera	87.6	86.2	1.4	4.5	7.9	2510
Illumina Arthropoda	63.3	62.2	0.7	23.9	12.8	1013
Hi-C Arthopoda	85.1	84.1	1.0	9.3	5.6	1013

The TPB has 17 chromosome pairs [38] and the 17 largest scaffolds with lengths of more than 1 Mb might represent the 17 chromosomes in the TPB. The GC percentage of 42.7% is higher than the pea aphid (29.6%) and honeybee (38.8%). Flow cytometry analysis of tissue from the heads of male and female TPB resulted in a genome size estimate of 816.6 +/- 2.6 Mb and 869.1 +/- 4.3 Mb, respectively, which is larger than the currently assembled reference assembly (Supplementary Fig. S2).

High repetitive content in the genome may have substantially reduced the genome size by the collapse of repetitive regions during the assembly process. We applied two approaches to identify repeats in the genome. The TPB genome assembly was searched for known repeat families in the order *insecta* present in the DFAM 2.4 database [39] (Supplementary data Table S1) but this resulted in the annotation of only 3.8% of the genome. RepeatModeler (http://www.repeatmasker.org/) identified 4281 RepeatScout/RECON families and 99 L repeat families with primarily Gypsy/DIRS1 elements. All annotations are available at the AgriVectors portal [34] public database.

Public databases currently list 2,191 and 1,552 nucleotide and protein sequences, respectively, for TPB. In addition, 8 Bioprojects, 21 Biosamples, and 17 population sets are available on the National Center for Bioinformatics (NCBI) database. Four of the eight Bioprojects were submitted by the USDA ARS Southern Insect Management Research Unit, including the TPB genome projects (PRJNA589321 and PRJNA685878) and three transcriptomics projects. We have published RNASeq data from the gut and salivary glands of TPB [26] and two other partial transcriptomes of TPB have been published previously [29, 40]. A high-quality genome with chromosome size scaffolds will facilitate the development of universal markers for mapping genomic loci associated with host selection, insecticide resistance, and population genomic studies. A chromosomal-length genome with annotations from NCBI will provide an official gene set to identify isoforms and study differential gene expression under various physiological conditions such as response to pesticides. The mapping of genomic DNA sequences to the published mitochondrial genome (accession: NC_021975) of TPB from the northern USA identified 34 nucleotide substitutions and three insertions in the protein-coding, rRNA, and tRNA genes of the mitochondrial DNA sequences of TPB from Mississippi. All variant positions, except five single nucleotide variants, were homozygous in southern TPB population.

Filtering of mapped reads identified 842,044 SNPs that were heterozygous in the reads mapped to the largest 18 scaffolds. Flanking sequences, allele-specific primers, and locus-specific primers developed for the manually selected SNPs are shown in the supplementary data Table S2.

Combined genomic and transcriptomic data (RNASeq + gDNA + BAC = 3,335,989,518 reads) will facilitate identifying non-transcribed genomic regions and regulatory sequences influencing gene expression. In addition, minor effect genes that are coregulated with major effect genes can be identified using expression profiles and gene coregulatory network analysis [41].

## Methods

TPB collected from field locations in Stoneville, MS were mated as single pairs to obtain progeny that were used to establish a colony inbred for five generations. DNA extracted from adult females from the inbred colony was submitted to Dovetail Genomics (Scotts Valley, CA) for library construction and genome sequencing. Illumina paired-end short reads (2 × 150 bp) were generated from a Chicago library made from TPB genomic DNA. Sequencing adapters and low-quality reads were removed before assembly using Trimmomatic [42]. All bases with quality scores lower than Q20 were removed from the leading and trailing ends and the middle of the reads.

A Dovetail Omni-C library was prepared as described in Saha et al. 2022 [43]. Briefly, chromatin was fixed in the nucleus by immersing the tissues in formaldehyde. Ends of DNAse I digested chromatin were repaired followed by ligation to a biotinylated bridge adapter. The adapter containing ends were proximity ligated and the crosslinks were reversed before the DNA was purified. Biotin not internal to ligated fragments were removed and the sequencing libraries containing Illumina-compatible adapters were generated using NEBNext Ultra reagents. Streptavidin beads were used to isolate biotin-containing DNA fragments and each library was PCR enriched. Illumina HiSeqX platform was used to sequence the libraries to approximately 30x coverage. HiRise, a pipeline specifically designed to scaffold initial genome assemblies using proximity ligation sequence data was used to generate final scaffolds using initial assembly and OmniC reads [44].

BUSCO version 5.2.2 was used to evaluate genome completeness [37]. Dfam TE tools docker container (version 1.4) of the RepeatModeler (https://github.com/Dfam-consortium/TETools) was used to annotate repeats. RepeatMasker and RepeatClassifier Version 2.0.2 (http://www.repeatmasker.org/) was used to classify the repeat types in the TPB genome. Dfam 3.4 database was used for repeat classification [39].

A published mitochondrial genome of TPB (accession: NC_021975) [45] was used as the reference to map 2,723,838,186 Illumina short reads generated by sequencing initial shotgun libraries and the Hi-C library using CLC Genome WorkBench (Qiagen, Redwood City, CA, USA). Variant analysis was performed on the mapped reads to identify single nucleotide polymorphisms and indels between the reference and the reads. Single nucleotide polymorphisms (SNP) were identified by filtering variants in Illumina reads mapped to the eighteen largest scaffolds using the variant filtering function in CLC Genome Workbench. SNPs with at least 60 mapped reads with greater than 30% heterozygosity and coverage greater than 200 were filtered and exported. A set of 96 SNPs representing 18 largest scaffolds were manually selected to develop an SNP assay panel.

Limitations: Proprietary methods developed by a service provider to prepare Genomic DNA library preparation and assembly are not publicly available. Difference between the genome size estimated by flow cytometry and the size of the assembled genome may needs to be corrected using long read technology.

## Electronic supplementary material

Below is the link to the electronic supplementary material.

Supplementary Material 1

Supplementary Material 2

## Data Availability

All raw sequencing data and assemblies have been submitted to NCBI BioProject: PRJNA685878.
