# Supplementary material for: A chromosome scale assembly of the tarnished plant bug, Lygus lineolaris (Palisot de Beauvois), genome
Source: BMC Res Notes. 2023 Jun 27;16:125. doi: 10.1186/s13104-023-06408-w (PMC10303854; doi:10.1186/s13104-023-06408-w)
Supplement: Supplementary file 1 — Supplementary Material 1 [file 13104_2023_6408_MOESM1_ESM.docx]

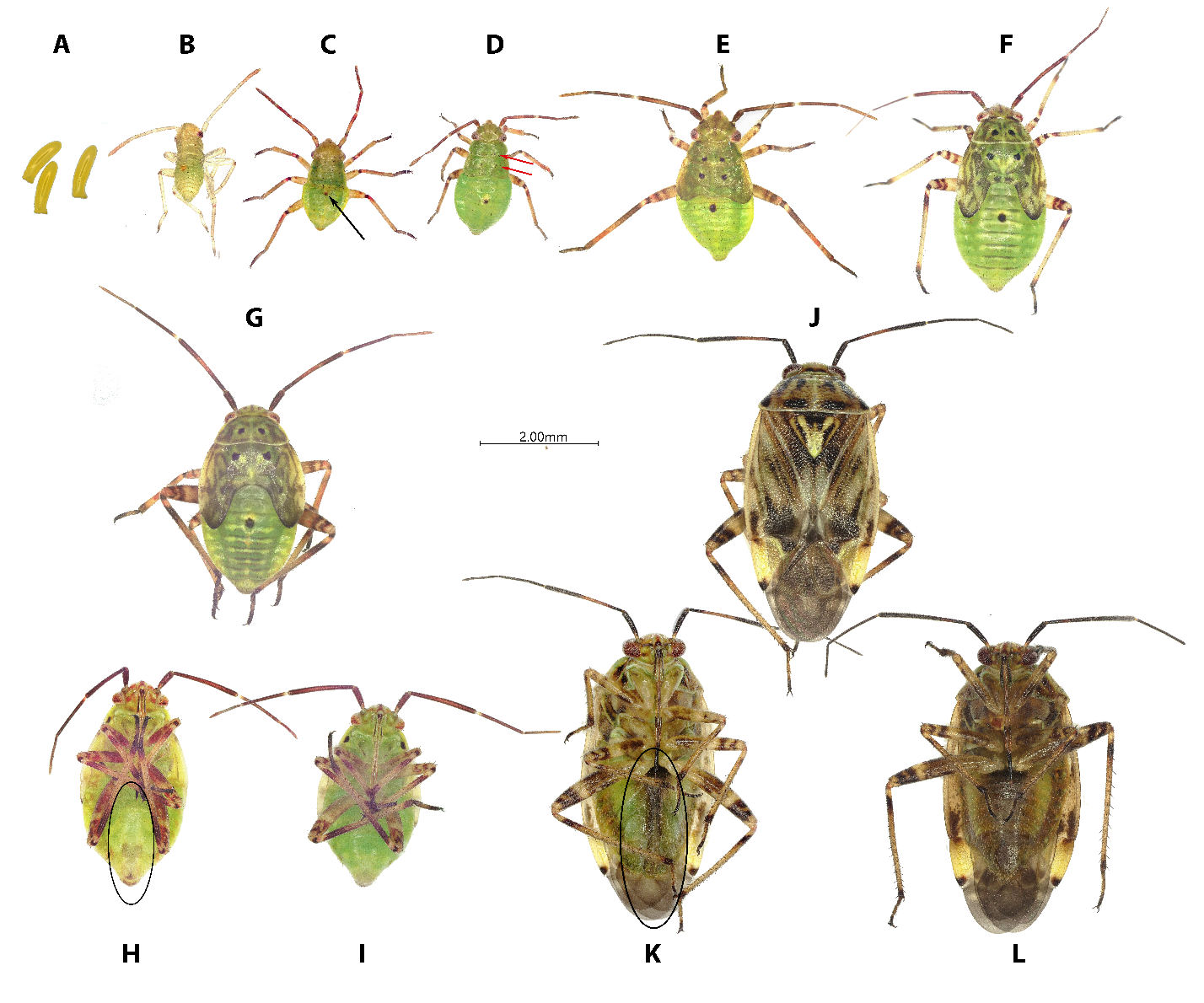
Figure S1. Different life stages of *Lygus lineolaris*. A. one-day old embryos. B. newly hatched first instar; C. one-day old first instar. A black arrow shows the dark spot in the abdomen; D. second instar with four thoracic spots (red arrows); E. third instar with prominent thoracic spots; F. fourth instar with wing pads extending in line with the abdominal spot; G. top view of a fifth instar with wing pads extending just below the abdominal spot; H. underside of a fifth instar female with recognizable ovipositor (oval), I. underside of a fifth instar male, J. top view of an adult male, K. underside of an adult female with prominently visible ovipositor (oval), and L­­­­. underside of an adult male.


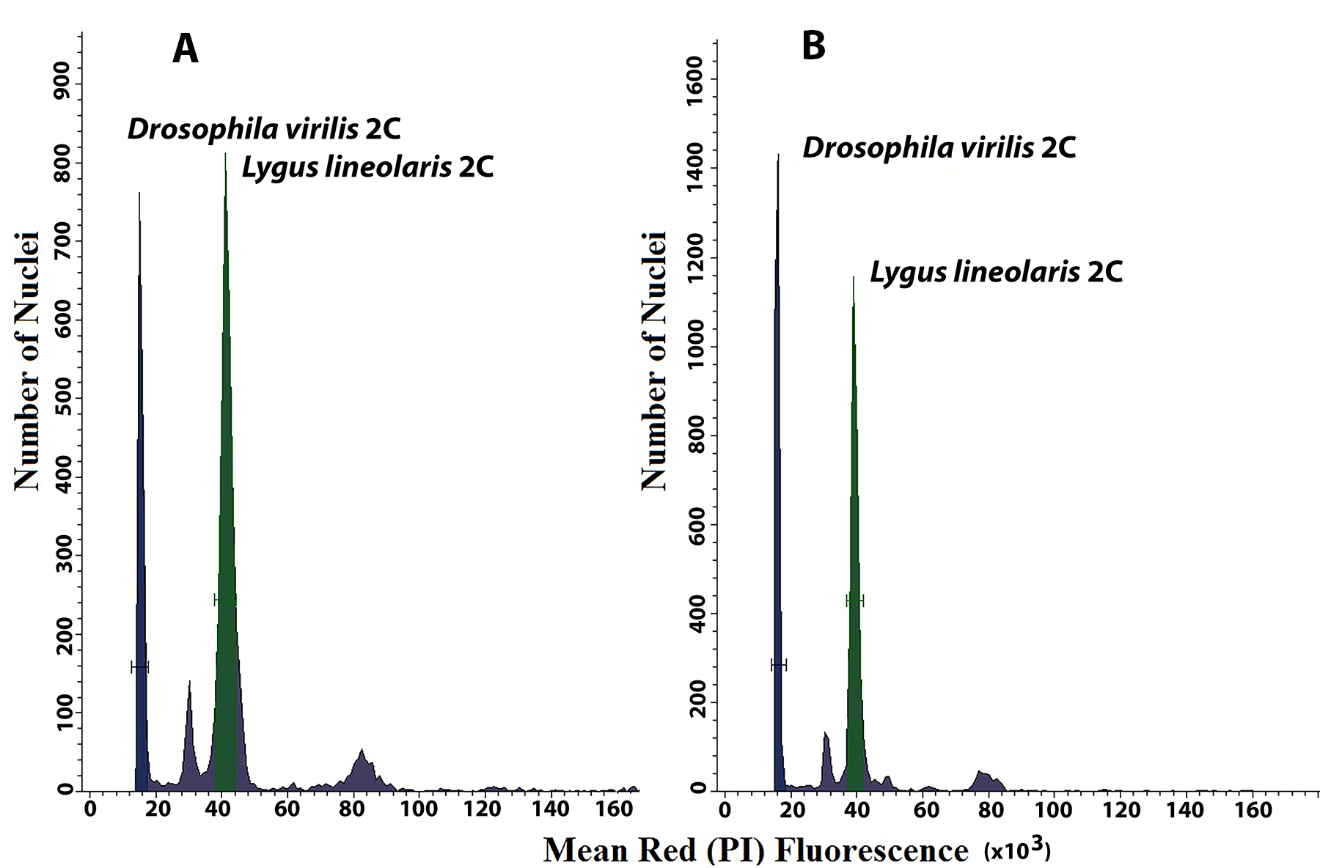


Figure S2. Flow cytometric estimation of *Lygus lineolaris* female (A) and male (B)genome sizes. *Drosophila virilis* nuclei were used as the standard.

Table S1. Different repeat families, the number of elements, the total length, and the percentage of each repeat type identified in *Lygus lineolaris* genome. RepeatMasker version 4.1.2-p1 was run in sensitive mode with rmblastn version 2.11.0+ and FamDB: CONS-Dfam_3.4.

| Type of repeat | Number of elements | Length occupied (bp) | % of sequence |
| --- | --- | --- | --- |
| Retroelements | 27635 | 4,615,344 | 0.77 |
| SINEs: | 7 | 400 | 0.00 |
| Penelope | 47 | 7,308 | 0.00 |
| LINEs: | 1992 | 313,683 | 0.05 |
| CRE/SLACS | 0 | 0 | 0.00 |
| L2/CR1/Rex | 562 | 48,333 | 0.01 |
| R1/LOA/Jockey | 167 | 18,649 | 0.00 |
| R2/R4/NeSL | 10 | 606 | 0.00 |
| RTE/Bov-B | 505 | 116,747 | 0.02 |
| L1/CIN4 | 0 | 0 | 0.00 |
| LTR elements | 25636 | 4,301,261 | 0.72 |
| BEL/Pao | 207 | 38,540 | 0.01 |
| Ty1/Copia | 180 | 45,650 | 0.01 |
| Gypsy/DIRS1 | 25233 | 4,214,739 | 0.70 |
| Retroviral | 0 | 0 | 0.00 |
|  |  |  |  |
| DNA transposons | 8990 | 1,249,837 | 0.21 |
| hobo-Activator | 2100 | 216,106 | 0.04 |
| Tc1-IS630-Pogo | 4434 | 737,469 | 0.12 |
| En-Spm | 0 | 0 | 0.00 |
| MuDR-IS905 | 0 | 0 | 0.00 |
| PiggyBac | 17 | 1,802 | 0.00 |
| Tourist/Harbinger | 0 | 0 | 0.00 |
| Other (Mirage, P-element, Transib) | 1 | 56 | 0.00 |
|  |  |  |  |
| Rolling-circles | 1836 | 125,613 | 0.02 |
|  |  |  |  |
| Unclassified: | 59182 | 6,381,207 | 1.06 |
|  |  |  |  |
| Total interspersed repeats |  | 12,246,388 | 2.04 |
|  |  |  |  |
| Small RNA: | 948 | 85,290 | 0.01 |
|  |  |  |  |
| Satellites: | 348 | 14,077 | 0.00 |
| Simple repeats: | 147900 | 5,737,020 | 0.96 |
| Low complexity: | 18280 | 891,808 | 0.15 |
